# The role of procalcitonin in predicting risk of mechanical ventilation and mortality among moderate to severe COVID-19 patients

**DOI:** 10.1186/s12879-022-07362-x

**Published:** 2022-04-15

**Authors:** Cher Wei Twe, Delton Kah Yeang Khoo, Kian Boon Law, Nur Sabreena binti Ahmad Nordin, Subashini Sathasivan, Kah Chuan Lim, Sharifah Khairul Atikah, Syarifah Nurul Ain bt Syed Badaruddin, Suresh Kumar Chidambaram

**Affiliations:** 1 Medical Department, Hospital Sungai Buloh, Ministry of Health, Sungai Buloh, Malaysia; 2 Digital Health Research and Innovation, Institute for Clinical Research, National Institutes of Health, Ministry of Health, Shah Alam, Malaysia; 3 Ophthalmology Department, Hospital Sungai Buloh, Ministry of Health, Sungai Buloh, Malaysia; 4 Pathology Department, Hospital Sungai Buloh, Ministry of Health, Sungai Buloh, Malaysia; 5 Clinical Research Center, Hospital Sungai Buloh, Ministry of Health, Sungai Buloh, Malaysia

**Keywords:** COVID-19, SARS-CoV-2, Risk factor, Procalcitonin, Mechanical ventilation, Mortality

## Abstract

**Background:**

Serum procalcitonin (PCT) has become an emerging prognostic biomarker of disease progression in patients with COVID-19. This study aims to determine the optimal cut-off value of PCT with regards to important clinical outcomes, especially for mechanical ventilation and all-cause mortality among moderate to severe COVID-19 patients in Malaysia.

**Methods:**

A total of 319 moderate to severe COVID-19 patients hospitalized at the National Referral Hospital in December 2020 were included in the study retrospectively. Demographics, comorbidities, the severity of COVID-19 infection, laboratory and imaging findings, and treatment given were collected from the hospital information system for analysis. The optimal cut-point values for PCT were estimated in two levels. The first level involved 276 patients who had their PCT measured within 5 days following their admission. The second level involved 237 patients who had their PCT measured within 3 days following their admission. Further, a propensity score matching analysis was performed to determine the adjusted relative risk of patients with regards to various clinical outcomes according to the selected cut-point among 237 patients who had their PCT measured within 3 days.

**Results:**

The results showed that a PCT level of 0.2 ng/mL was the optimal cut-point for prognosis especially for mortality outcome and the need for mechanical ventilation. Before matching, patients with PCT ≥ 0.2 ng/mL were associated with significantly higher odds in all investigated outcomes. After matching, patients with PCT > 0.2 ng/mL were associated with higher odds in all-cause mortality (OR: 4.629, 95% CI 1.387–15.449, p = 0.0127) and non-invasive ventilation (OR: 2.667, 95% CI 1.039–6.847, p = 0.0415). Furthermore, patients with higher PCT were associated with significantly longer days of mechanical ventilation (p = 0.0213). There was however no association between higher PCT level and the need for mechanical ventilation (OR: 2.010, 95% CI 0.828–4.878, p = 0.1229).

**Conclusion:**

Our study indicates that a rise in PCT above 0.2 ng/mL is associated with an elevated risk in all-cause mortality, the need for non-invasive ventilation, and a longer duration of mechanical ventilation. The study offers concrete evidence for PCT to be used as a prognostication marker among moderate to severe COVID-19 patients.

## Background

Since the outbreak of coronavirus disease 2019 (COVID-19) in December 2019 in Wuhan, China, the COVID-19 has rapidly spread across the world and was declared as a “Public Health Emergency of International Concern” on 30th January 2020. Then, it emerged as an unanticipated threat to global health and led the World Health Organisation (WHO) to further declare COVID-19 as a pandemic on 11th March 2020 [1]. As of 18th September 2021, there are more than 226 million confirmed COVID-19 cases with 4.7 million deaths reported globally. Malaysia has reported 2,049,750 cases with 22,355 deaths on the same day [2].

The clinical spectrum of COVID-19 ranges from asymptomatic to symptomatic severe disease with multiorgan involvement and failure requiring intensive care (ICU) admission and mechanical ventilation [3]. The broad clinical spectrum and highly variable clinical course among different COVID-19 patients have introduced great challenges in predicting the disease progression and outcome [4]. Laboratory biomarkers have always been very useful in day-to-day clinical practice to guide disease management and treatment decisions, especially in the management of infectious diseases like the COVID-19. Hence, ever since the start of the outbreak, the use of laboratory biomarkers including C-reactive protein (CRP), d-dimer, lactate dehydrogenase (LDH), and procalcitonin (PCT) to predict disease progression and severity have been explored and investigated extensively [1, 5].

Procalcitonin (PCT) is a 116-amino-acid peptide that shares a common molecular structure with the prohormone of calcitonin. It was first discovered in humans in 1981 by Allison et al. but its clinical use was never really explored until 1993 when Assicot et al. first suggested a positive association between elevated serum PCT and bacterial infection and sepsis [6, 7]. In today’s clinical practice, serum PCT has been increasingly instrumental in antibiotic stewardship and the diagnosis and management of sepsis due to bacterial infection [8].

Since the beginning of the COVID-19 pandemic, multiple observational studies and meta-analyses have been done to look into the utility of serum PCT level as a biomarker of clinical deterioration among COVID-19 patients and have shown encouraging results with various optimal PCT cut-points suggested [9–11]. A single-center retrospective study done in Wuhan, China showed that a serum PCT level of more than 0.2 ng/mL was found in severe and critical COVID-19 patients [12]. Another retrospective study of 1099 patients reported that a PCT of more than 0.5 ng/dL was associated with increased severity of COVID-19 infection [13]. Theoretically, PCT, an acute phase peptide, is released in response to proinflammatory cytokines like IL-1β, IL-6, and TNF-α typically induced by bacterial infection and are not seen in viral infections [14]. However, it was found that these pro-inflammatory cytokines are also raised in COVID-19 infection, especially among severe diseases, which makes serum PCT a promising biomarker of clinical deterioration among COVID-19 patients [15].

This study aims to investigate the optimal cut-point of PCT and its relationship with regards to various clinical outcomes, especially in all-cause mortality and the need for mechanical ventilation among moderate to severe COVID-19 patients requiring hospitalization and treatment.

## Methods

### Study design and setting

A total of 319 patients who were diagnosed with moderate to severe COVID-19 pneumonia and admitted to Sungai Buloh Hospital in December 2020 were included and studied retrospectively. Demographic information, including age, gender, race, concomitant medical illnesses, the severity of COVID-19 infection, laboratory and imaging findings, and treatment given were retrieved from the hospital information system for analysis. The study included category 4 and 5 adult patients above 18 years old with a diagnosis of COVID-19 confirmed via polymerase chain reaction (PCR) test (Table 1). Patients were excluded if no reported PCT values.

**Table 1:** Classification of COVID-19 infection severity

Clinical stage	Severity	Description
1	Mild	Asymptomatic - Only RT-PCR test positive
2		Symptomatic, but no pneumonia - Upper respiratory tract (URT) symptoms (e.g., pharyngeal congestion, sore throat, cough, or fever) - Other symptoms, like vomiting, diarrhea, abdominal pain, myalgia, loss of smell/taste
3	Moderate	Symptomatic, pneumonia, but no hypoxemia
4	Severe	Symptomatic, pneumonia, requiring supplemental oxygen OR New requirement of supplemental oxygen or increased requirement from baseline without need for non-invasive or invasive ventilation
5	Critical	Critically Ill with multiorgan involvement OR New or increased need for non-invasive or invasive ventilation, including high flow nasal cannula

**Fig. 1:**
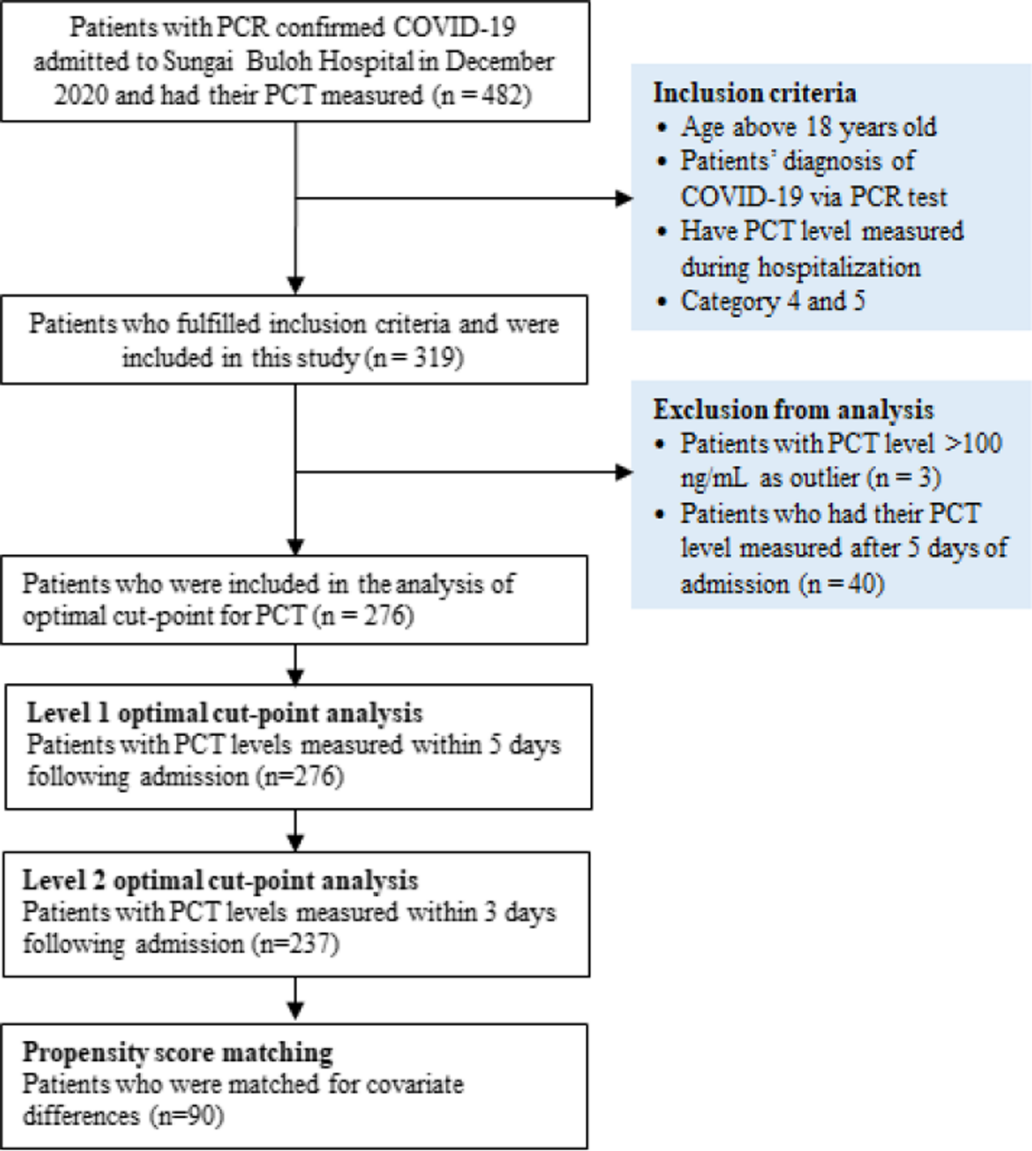
Flowchart of patient recruitment

The majority of patients (87.1%) had their PCT levels measured within 5 days following their admission. As PCT levels may reduce over time after receiving treatment, we included patients with their PCT levels measured within 5 days and 3 days for the analysis of the optimal cut-point. Hence, the analysis of the optimal cut-point for PCT was performed in two levels. The first level involved 276 patients with their PCT levels measured within 5 days following admission and the second level involved 237 patients with their PCT levels measured within 3 days following admission. Besides, the study excluded patients with extremely high PCT levels (> 100 ng/mL) to avoid overly skewed findings. Clinical outcomes included in the analysis for the optimal PCT cut-point were all-cause mortality, mechanical ventilation, the occurrence of thrombotic events, ICU admission, and bacterial infection. Figure 1 summarizes the patient recruitment process.

In the study, COVID-19 infection was classified according to the Malaysian Clinical Practice Guideline for Management of COVID-19 [3]. The guideline classifies COVID-19 severity into five clinical stages as shown in Table 1. Stage 1 and 2 consist of patients with asymptomatic infection or symptomatic infection with no pneumonia. Patients in these two categories are considered to have mild disease. Patients with stage 3 disease have lung involvement or pneumonia, but have not experienced hypoxemia, while stage 4 and 5 consist of critically ill patients requiring oxygen support and often intensive care.

### Analysis of PCT

In Sungai Buloh Hospital, serum PCT was measured by SIEMENS ATELLICA IMMUNOASSAY (IM). Atellica IM BRAHMS PCT assay is a 2-site sandwich immunoassay using direct chemiluminescent technology that uses 3 mouse monoclonal antibodies specific for PCT. The first antibody, in the Lite Reagent, is a mouse monoclonal anti‑PCT antibody labeled with acridinium ester. The second and third antibodies, in the Ancillary Reagent, are mouse monoclonal anti‑PCT antibodies labeled with fluorescein. The immunocomplex formed with PCT is captured with mouse monoclonal anti-fluorescein antibody coupled to paramagnetic particles in the Solid Phase. This 18-min sandwich immunoassay with a measuring range of 0.03 to 50.00 ng/mL, is aligned to the B·R·A·H·M·S PCT sensitive KRYPTOR^®^ assay [16].

### Statistical analysis

Demographics and clinical characteristics were summarized in Table 2 for overall 319 patients, 276 and 237 patients who had their PCT measured within 5 days and 3 days following their admission. The optimal PCT cut-points were estimated using the receiver operative characteristic (ROC) method in two levels. In the first level, we included 276 patients who had their PCT levels measured within 5 days following their admission. In the second level, we included 237 patients who had their PCT levels measured within 3 days following their admission. The optimal cut-point was determined with priority on all-cause mortality and the need for mechanical ventilation. In further analysis, patients with their PCT levels measured within 3 days following admission were divided into two groups according to the selected cut-point. Patients with PCT levels above the cut-point were assumed to have a higher risk for inferior clinical outcomes, and vice versa. We compared and tested the differences of demographics, clinical characteristics, baseline laboratory findings, and co-morbidity profile between the two groups to identify covariates for adjustment in the propensity score matching analysis. The odds ratio (OR) was calculated and reported for various clinical outcomes with regards to the selected cut-point for PCT before and after the matching analysis. Independent t-test and Mann–Whitney U test were used to test differences of continuous variables between two groups, while Chi-squared test and Fisher’s exact test were used to assess categorical variables. All statistical tests were performed at two-sided 5% significance level.

**Table 2:** Demographics and clinical characteristics of the study cohort

Characteristics	Overall n (%)	First level n (%)	Second level n (%)
Total	319 (100.0)	276 (100.0)	237 (100.0)
Age, years: Mean (SD)	56 (13.6)	54.8 (13.7)	55.0 (13.7)
Gender: Female	111 (34.8)	102 (37.0)	84 (35.4)
Race			
Malay	168 (52.7)	147 (53.3)	122 (51.5)
Chinese	68 (21.3)	54 (19.6)	46 (19.4)
Indian	52 (16.3)	48 (17.4)	44 (18.6)
Others	31 (9.7)	27 (9.8)	25 (10.5)
Comorbidities profile			
Hypertension: Yes	179 (56.1)	158 (57.2)	135 (57.0)
Chronic cardiac disease: Yes	64 (20.1)	54 (19.6)	46 (19.4)
Chronic pulmonary disease: Yes	10 (3.1)	8 (2.9)	7 (3.0)
Asthma: Yes	14 (4.4)	13 (4.7)	10 (4.2)
Diabetes mellitus: Yes	147 (46.1)	128 (46.4)	110 (46.4)
Pre-existing renal disease: Yes	73 (22.9)	56 (20.3)	46 (19.4)
Chronic liver disease: Yes	3 (0.9)	2 (0.7)	2 (0.8)
Dementia: Yes	3 (0.9)	3 (1.1)	2 (0.8)
Chronic neurological conditions: Yes	16 (5.0)	13 (4.7)	10 (4.2)
Connective tissue disease: Yes	7 (2.2)	7 (2.5)	5 (2.1)
HIV/AIDS: Yes	2 (0.6)	1 (0.4)	1 (0.4)
Malignancy: Yes	11 (3.4)	9 (3.3)	8 (3.4)
Current smoking: Yes	9 (2.8)	5 (1.8)	4 (1.7)
Obesity: Yes	17 (5.3)	17 (6.2)	16 (6.8)
Others: Yes	81 (25.4)	67 (24.3)	51 (21.5)
COVID-19 severity when procalcitonin first sent			
Category 4	254 (79.6)	217 (78.6)	181 (76.4)
Category 5	65 (20.4)	59 (21.4)	56 (23.6)
4C mortality score			
0–3 (low-risk in-hospital mortality)	16 (5.0)	15 (5.4)	13 (5.5)
4–8 (intermediate-risk in-hospital mortality)	117 (36.7)	105 (38.0)	87 (36.7)
9–14 (high-risk in-hospital mortality)	145 (45.5)	124 (44.9)	107 (45.1)
15–21 (very high-risk in-hospital mortality)	41 (12.8)	32 (11.6)	30 (12.7)
Steroid use: Yes	297 (93.1)	257 (93.1)	219 (92.4)
Bloodstream infection: Yes	35 (11.0)	28 (13.0)	25 (13.4)
Clinical outcomes			
ICU admission: Yes	143 (44.8)	129 (46.7)	120 (50.6)
NIV use: Yes	65 (20.4)	60 (21.7)	57 (24.1)
Duration of NIV use, days, median (IQR)	3 (1.8, 5.3)	3 (1.8, 5.3)	3 (1, 5)
Mechanical ventilation: Yes	101 (31.7)	89 (32.2)	85 (35.9)
Duration of mechanical ventilation use, days, median (IQR)	6 (4, 12)	7 (4, 12)	7 (4, 12)
Organizing pneumonia			
Yes	135 (42.3)	118 (42.8)	102 (43.0)
No	26 (8.2)	21 (7.6)	19 (8.0)
CT scan not done	158 (49.5)	137 (49.6)	116 (48.9)
Thrombotic event: Yes	83 (26.0)	69 (25.0)	62 (26.2)
All-cause mortality: Yes	71 (22.3)	59 (21.4)	55 (23.2)
Due to severe COVID-19 pneumonia	48 (67.6)	43 (72.9)	39 (70.9)
Due to thrombotic event	8 (11.3)	7 (16.3)	7 (17.9)
Due to comorbid	7 (9.8)	4 (9.3)	4 (10.3)
Due to bacterial infections	8 (11.3)	5 (11.6)	5 (12.8)
Bacteremia	3 (4.2)	1 (2.3)	1 (2.6)
Intra-abdominal infection	1 (1.4)	0 (0)	0 (0)
Necrotizing fasciitis	1 (1.4)	1 (2.3)	1 (2.6)
Non-specified site	1 (1.4)	0 (0)	0 (0)
Non-bacteremia	5 (7.0)	4 (9.3)	4 (10.3)
Pulmonary infection	2 (2.8)	1 (2.3)	1 (2.6)
Intra-abdominal infection	1 (1.4)	1 (2.3)	1 (2.6)
Non-specified site	2 (2.8)	2 (4.7)	2 (5.1)

Column percentages are reported in parenthesis for categorical variables; mean and standard deviation are reported for continuous variable age; median and interquartile range are reported for continuous variables duration of non-invasive ventilation and mechanical ventilation

*CT* computed tomography, *HIV/AIDS* human immunodeficiency virus/acquired immunodeficiency syndrome, *ICU* intensive care unit, *NIV* non-invasive ventilation

In propensity score matching analysis, patients were matched for covariates that were statistically different. Baseline laboratory parameters and vital signs were not included in the matching analysis as they were not inherited but clinical manifestations of COVID-19 infection or complications. Propensity score matching was done based on the nearest-neighbor method within a caliper width equal to 0.25 times the SD of the logit of the calculated propensity score. All analyses were performed in IBM SPSS version 26.0 for Windows and R version 4.1.0 with packages such as “cutpointr” and “MatchIt” [17–19].

## Results

Demographics, clinical characteristics, and clinical outcomes of patients were summarized in Table 2 according to the overall cohort, first and second level cohorts. Even with the exclusion of patients due to delayed measurement of PCT, first and second level cohorts were still similar to the overall cohort in terms of clinical characteristics, comorbidities profile, and clinical outcomes.

### Determination of the optimal cut-point

In the determination of optimal cut-points for PCT, we included clinical outcomes, such as all-cause mortality, mechanical ventilation, thrombotic events, ICU admissions, and bacterial infection among moderate to severe COVID-19 patients. Tables 3 and 4 present various performance indexes and optimal cut-points for PCT with regards to the aforementioned clinical outcomes based on PCT levels measured within 5 days and 3 days following admission.

**Table 3:** Optimal cut-off PCT values (within 5 days from admission)

Cut-point and indexes	All-cause mortality	Mechanical ventilation	Thrombotic events	ICU admission	Bacterial infection culture
Number of patients	276	276	276	276	216
Positive cases	59	89	69	129	28
Negative cases	217	187	207	147	188
AUC	0.7741	0.7773	0.6666	0.7223	0.6444
Optimal cut-point (ng/mL)	0.20	0.33	1.21	0.09	1.2
Accuracy	0.6703	0.7391	0.7536	0.6848	0.7454
Sensitivity	0.8136	0.6742	0.4203	0.7442	0.5357
Specificity	0.6313	0.7701	0.8647	0.6327	0.7766
Precision	0.3750	0.5825	0.5088	0.6400	0.2632
TP	48	60	29	96	15
FN	11	29	40	33	13
FP	54	43	28	43	42
TN	93	144	179	144	146

*TP* true positive, *FN* false negative, *FP* false positive, *TN* true negative, *AUC* area under curve

**Table 4:** Optimal cut-off PCT values (within 3 days from admission)

Cut-point and Indexes	All-cause mortality	Mechanical ventilation	Thrombotic events	ICU admission	Bacterial infection culture
Number of patients	237	237	237	237	186
Positive cases	55	85	62	120	25
Negative cases	182	152	175	117	161
AUC	0.7812	0.7676	0.6711	0.7365	0.6465
Optimal cut-point (ng/mL)	0.20	0.21	1.21	0.07	1.25
Accuracy	0.6667	0.7046	0.7468	0.7046	0.7419
Sensitivity	0.8364	0.7528	0.4355	0.8333	0.5600
Specificity	0.6154	0.6776	0.8571	0.5726	0.7702
Precision	0.3966	0.5663	0.5192	0.6667	0.2745
TP	46	64	27	100	14
FN	9	21	35	20	11
FP	70	49	25	50	37
TN	112	103	150	67	124

*TP* true positive, *FN* false negative, *FP* false positive, *TN* true negative, *AUC* area under curve

For PCT levels measured within 5 days following admission, the optimal cut-points for PCT were found to be 0.20 ng/mL for all-cause mortality, 0.33 ng/mL for mechanical ventilation, 1.21 ng/mL for thrombotic events, 0.09 ng/mL for ICU admission dan 1.2 ng/mL for bacterial infection (Table 3). Highest sensitivity was found in all-cause mortality (0.8136) for PCT cut-point of 0.20 ng/mL. For PCT levels measured within 3 days following admission, the optimal cut-points for PCT were found to be 0.20 ng/mL for all-cause mortality, 0.21 ng/mL for mechanical ventilation, 1.21 ng/mL for thrombotic events, 0.07 ng/mL for ICU admission dan 1.25 ng/mL for bacterial infection (Table 4). Highest sensitivity was also found in all-cause mortality (0.8364) for PCT cut-point of 0.20 ng/mL.

As 0.2 ng/mL appeared to be the optimal cut-point in all-cause mortality for PCT levels measured within 5 days and 3 days following admission, and in mechanical ventilation for PCT levels measured within 3 days following admission, therefore, 0.2 ng/mL was selected as the final PCT cut-point in the matching analysis using propensity scores. The PCT of 0.2 ng/mL represented the optimal cut-point for the two clinically important outcomes, all-cause mortality and mechanical ventilation, especially for moderate to severe COVID-19 patients with PCT measured within 3 days following admission.

### Propensity scores matching analysis

To assess the prognostic value of the optimal cut-point, the analysis focused on those who had their PCT levels measured within 3 days following admission. Moreover, the selected cut-point of 0.2 ng/mL was optimal for all-cause mortality and mechanical ventilation among those with PCT levels measured within 3 days following admission. Table 5 summarizes the covariate differences according to the selected optimal cut-point for PCT among 237 patients. Significant differences were identified for age (p = 0.0122), COVID-19 disease stage (p < 0.0001), days of illness before admission (p = 0.0036), SPO_2_ under room temperature (RA) (p = 0.0005), Glasgow Coma Scale (GCS) (p < 0.0001), urea (p < 0.0001), C-reactive protein (CRP) (p < 0.0001), white blood cells count (WBC) (p = 0.0003), absolute neutrophil count (ANC) (p < 0.0001), number of comorbidities (p < 0.0001), presence of hypertension (p = 0.0007), chronic cardiac diseases (p = 0.0018), diabetes mellitus (DM) (p = 0.0036), pre-existing renal disease (p < 0.0001) and malignancy (p = 0.0327).

**Table 5:** Demographics, clinical characteristics and baseline laboratory findings, and comorbidity profile of COVID-19 patients according to procalcitonin level

Variables	Category	PCT < 0.2 (ng/mL) (n = 121)	PCT ≥ 0.2 (ng/mL) (n = 116)	p-value
Demographics				
Gender	Female	42 (34.7)	42 (36.2)	0.8097
Age (years)	Mean (SD)	52.8 (12.6)	57.3 (14.4)	0.0122
Age groups (years)	< 50	45 (37.2)	33 (28.4)	0.0945
	50–59	38 (31.4)	28 (24.1)	
	60–60	23 (19.0)	34 (29.3)	
	≥ 70	15 (12.4)	21 (18.1)	
Ethnicity	Malay	60 (62.3)	62 (53.4)	0.0675
	Chinese	22 (18.2)	24 (20.7)	
	Indian	20 (16.5)	24 (20.7)	
	Others	19 (15.7)	6 (5.2)	
Clinical characteristics and baseline laboratory				
Severity of disease	Category 4	111 (91.7)	70 (60.3)	< 0.0001
	Category 5	10 (8.3)	46 (39.7)	
Days of illness	Mean (SD)	6.5 (3.0)	5.3 (3.1)	0.0036
SPO_2_ under RA (%)	< 92%	98 (81.0)	111 (95.7)	0.0005
	≥ 92%	23 (19.0)	5 (4.3)	
RR, bpm	< 20	13 (10.7)	14 (12.1)	0.2520
	20–29	93 (76.9)	79 (68.1)	
	≥ 30	15 (12.4)	23 (19.8)	
GCS	< 15	11 (9.1)	50 (43.1)	< 0.0001
	15	110 (90.9)	66 (56.9)	
Urea, mmol/dL	< 7	87 (71.9)	31 (26.7)	< 0.0001
	7–14	29 (24.0)	31 (26.7)	
	> 14	5 (4.1)	54 (46.6)	
CRP, mg/dL	< 5	49 (40.5)	8 (6.9)	< 0.0001
	5–9.9	31 (25.6)	69 (59.5)	
	≥ 10	41 (33.9)	39 (33.6)	
WBC (× 10^12^/L)	Mean (SD)	8.56 (3.88)	10.90 (5.80)	0.0003
ANC (× 10^9^/L)	Mean (SD)	6.75 (3.81)	9.26 (5.53)	< 0.0001
Comorbidity profile				
No. of comorbidity	0	67 (55.4)	33 (28.4)	< 0.0001
	1	42 (34.7)	33 (28.4)	
	≥ 2	12 (9.9)	50 (43.1)	
Hypertension	Yes	56 (46.3)	79 (68.1)	0.0007
Chronic cardiac disease (excluding HPT)	Yes	14 (11.6)	32 (27.6)	0.0018
Asthma	Yes	6 (5.0)	4 (3.4)	0.7491
Chronic pulmonary disease (excluding asthma)	Yes	3 (2.5)	4 (3.4)	0.7174
Diabetes Mellitus	Yes	45 (37.2)	65 (56.0)	0.0036
Pre-existing renal disease	Yes	5 (4.1)	41 (35.3)	< 0.0001
Stage 2		2 (40.0)	0	
Stage 3		0	6(14.6)	
Stage 4		2 (40.0)	8 (19.5)	
Stage 5		1 (20.0)	27 (65.9)	
HIV/AIDS	Yes	1	0	n.a
Malignancy	Yes	1 (0.8)	7 (6.0)	0.0327
Smoking	Yes, current	1 (0.8)	3 (2.6)	n.a
Obesity (> 30 kg/m^2^)	Yes	7 (5.8)	9 (7.8)	0.5449
Chronic liver disease	Yes	0	2	n.a
Dementia	Yes	0	2	n.a
Chronic neurological disease	Yes	2 (1.7)	8 (6.9)	0.0555
Connective tissue disease	Yes	2 (1.7)	3 (2.6)	0.6783

*ANC* absolute neutrophil count, *CRP* C-reactive protein, *GCS* Glasgow coma scale, *HIV/AIDS* human immunodeficiency virus/acquired immunodeficiency syndrome, *PCT* procalcitonin, *RR* respiratory rate, *RA* room air, *SD* standard deviation, *WBC* white blood cell count

Table 6 presents the odds ratio (OR) with regards to various clinical outcomes based on the optimal cut-point of 0.2 ng/mL for PCT. Prior to matching, our analysis found that patients with PCT level above 0.2 ng/mL were associated with significantly higher risk in all-cause mortality (OR: 8.178, 95% CI 3.770–17.738, p < 0.0001), mechanical ventilation (OR: 5.861, 95% CI 3.229–10.637, p < 0.0001), non-invasive ventilation (OR: 2.898, 95% CI 1.541–5.541, p = 0.0010), ICU admission (OR: 4.166, 95% CI 2.422–7.166, p < 0.0001) and thrombotic events (OR: 2.158, 95% CI 1.190–3.915, p = 0.0013). Besides, patients with PCT levels above 0.2 ng/mL also experienced significantly longer days of mechanical ventilation (p < 0.0106) and length of hospital stay (p < 0.0001). Table 7 shows the mortality and causes of death before propensity score matching.

**Table 6:** Clinical outcomes before propensity score matching

Clinical outcomes	Category	PCT level (ng/mL)		OR (95% CI)	p-value
		PCT < 0.2 (n = 121)	PCT ≥ 0.2 (n = 116)		
Procalcitonin level	Mean (SD)	0.0674 (0.0331)	6.002 (11.556)	n.a	< 0.0001
All-cause mortality	Yes	9 (7.4)	46 (39.7)	8.178 (3.770–17.738)	< 0.0001
	No	112 (92.6)	70 (60.3)		
Mechanical ventilation	Yes	21 (17.4)	64 (55.2)	5.861 (3.229–10.637)	< 0.0001
	No	100 (82.6)	52 (44.8)		
Non-invasive ventilation	Yes	18 (14.9)	39 (33.6)	2.898 (1.541–5.541)	0.0010
	No	103 (85.1)	77 (66.4)		
ICU admission	Yes	41 (33.9)	79 (68.1)	4.166 (2.422–7.166)	< 0.0001
	No	80 (66.1)	37 (31.9)		
Thrombotic events	Yes	23 (19.0)	39 (33.6)	2.158 (1.190–3.915)	0.0113
	No	98 (81.0)	77 (66.4)		
Positive culture	Yes	68 (89.5)	93 (84.5)	0.643 (0.263–1.578)	0.3354
	No	8 (10.5)	17 (15.5)		
Days of mechanical ventilation	Median (IQR)	5 (4–6)	9 (5–12.3)	n.a	0.0106
Days of non-invasive ventilation	Median (IQR)	3 (2–4)	3 (1–6)		0.8071
Length of hospital stay, days	Median (IQR)	11 (9–14)	16 (11.3–20.8)	n.a	< 0.0001

*CI* confidence interval, *ICU* intensive care unit, *IQR* interquartile range, *MV* mechanical ventilation, *NIV* non-invasive ventilation, *OR* odds ratio, *PCT* procalcitonin

**Table 7:** Mortality and causes of death before propensity score matching

Mortality outcomes	Cases (%)
Total number of deaths	55
Cause of death	
Severe COVID-19 pneumonia	39 (70.9)
Bacterial infection	5 (9.1)
Bacteremia	1
Non-bacteremia	4
Thrombotic event	7 (12.7)
Comorbidity	4 (7.3)

As the relative risk of patients categorized based on the optimal cut-point could be confounded by covariates that were significantly different, a propensity score matching was performed with the aforementioned covariates to derive two comparable arms for further analysis. The propensity score matching eventually identified 90 patients with balanced demographics, clinical characteristics, comorbidities profile, and laboratory findings except for urea (p = 0.0139) and CRP (p < 0.0001) as shown in Table 8. Subsequent analyses showed that patients with PCT above 0.2 ng/mL were associated with a significantly higher risk in all-cause mortality (OR: 4.629, 95% CI 1.387–15.449, p = 0.0127), and marginally higher risk for non-invasive ventilation (OR: 2.667, 95% CI 1.039–6.847, p = 0.0415). Besides, patients with PCT above 0.2 ng/mL tended to experience longer days of mechanical ventilation (p = 0.0213) as summarized in Table 9. There was no significant association between PCT level and risk of mechanical ventilation (OR; 2.010, 95% CI 0.828–4.878, p = 0.1229) after being matched for covariate differences.

**Table 8:** Demographics, clinical characteristics and baseline laboratory findings and comorbidity profile of COVID-19 patients according to procalcitonin level after propensity score matching

Variables	Category	PCT < 0.2 (n = 45)	PCT ≥ 0.2 (n = 45)	p-value
Demographics				
Gender	Female	17 (37.8)	17 (37.8)	1.0000
Age	Mean (SD)	55.1 (11.2)	53.4 (13.1)	0.5070
Age groups	< 50	13 (28.9)	16 (35.6)	0.7838
	50–59	14 (31.1)	15 (33.3)	
	60–60	13 (28.9)	9 (20.0)	
	≥ 70	5 (11.1)	5 (11.1)	
Ethnicity	Malay	22 (48.9)	27 (60.0)	0.6236
	Chinese	9 (20.0)	7 (15.6)	
	Indian	8 (17.8)	8 (17.8)	
	Others	6 (13.3)	3 (6.7)	
Clinical characteristics and baseline laboratory				
Severity of disease	Category 4	35 (77.8)	37 (82.2)	0.5982
	Category 5	10 (22.2)	8 (17.8)	
Days of illness	Mean (SD)	5.7 (2.8)	5.6 (3.0)	0.8852
SPO_2_ under RA (%)	< 92%	38 (84.4)	42 (93.3)	0.1797
	≥ 92%	7 (15.6)	3 (6.7)	
RR, bpm	< 20	5 (11.1)	4 (8.9)	
	20–29	33 (73.3)	31 (68.9)	
	≥ 30	7 (15.6)	10 (22.2)	
GCS	< 15	9 (20.0)	11 (24.4)	0.6121
	15	36 (80.0)	34 (75.6)	
Urea, mmol/dL	< 7	24 (53.3)	20 (44.4)	0.0139
	7–14	17 (37.8)	10 (22.2)	
	> 14	4 (8.9)	15 (33.3)	
CRP, mg/dL	< 5	17 (37.8)	1 (2.2)	< 0.0001
	5–9.9	13 (28.9)	15 (33.3)	
	≥ 10	15 (33.3)	29 (64.4)	
WBC (× 10^12^/L)	Mean (SD)	9.79 (4.26)	10.69 (6.09)	0.4148
ANC (× 10^9^/L)	Mean (SD)	7.89 (4.32)	8.74 (5.82)	0.4328
Comorbidity profile				
No. of comorbidity	0	15 (33.3)	15 (33.3)	0.9654
	1	19 (42.2)	18 (40.0)	
	≥ 2	11 (24.4)	12 (26.7)	
Hypertension	Yes	24 (53.3)	27(60.0)	0.5234
Chronic cardiac disease (excluding HPT)	Yes	12 (26.7)	12 (26.7)	1.0000
Asthma	Yes	1 (2.2)	0	n.a
Chronic pulmonary disease (excluding asthma)	Yes	1 (2.2)	2 (4.4)	n.a
Diabetes mellitus	Yes	24 (53.3)	21 (46.7)	0.5271
Pre-existing renal disease	Yes	5 (11.1)	9 (20.0)	0.2447
Stage 2		2	0	
Stage 3		0	1	
Stage 4		2	0	
Stage 5		1	8	
HIV/AIDS	Yes	0	0	n.a
Malignancy	Yes	1 (2.2)	0	n.a
Smoking	Yes, current	0	2 (4.4)	n.a
Obesity (> 30 kg/m^2^)	Yes	2 (4.4)	4 (8.9)	0.6766
Chronic liver disease	Yes	0	1 (2.2)	
Dementia	Yes	0	0	
Chronic neurological disease	Yes	2 (4.4)	1 (2.2)	
Connective tissue disease	Yes	2 (4.4)	2 (4.4)	

*ANC* absolute neutrophil count, *CRP* C-reactive protein, *GCS* Glasgow Coma Scale, *HIV/AIDS* human immunodeficiency virus/acquired immunodeficiency syndrome, *PCT* procalcitonin, *RR* respiratory rate, *RA* room air, *SD* standard deviation, *WBC* white blood cell count

**Table 9:** Clinical outcomes after propensity score matching

Clinical outcomes	Category	PCT level		OR (95% CI)	p-value
		PCT < 0.2 (n = 45)	PCT ≥ 0.2 (n = 45)		
Procalcitonin level	Mean (SD)	0.068 (0.033)	4.849 (11.624)	n.a	0.0084
All-cause mortality	Yes	4 (8.9)	14 (31.1)	4.629 (1.387–15.449)	0.0127
	No	41 (91.1)	31 (68.9)		
Mechanical ventilation	Yes	12 (26.7)	19 (42.2)	2.010 (0.828–4.878)	0.1229
	No	33 (73.3)	26 (57.8)		
Non-invasive ventilation	Yes	9 (20.0)	18 (40.0)	2.667 (1.039–6.847)	0.0415
	No	36 (80.0)	27 (60.0)		
ICU admission	Yes	23 (51.1)	28 (62.2)	1.575 (0.681–3.648)	0.2886
	No	22 (48.9)	17 (37.8)		
Thrombotic events	Yes	10 (22.2)	15 (33.3)	1.750 (0.686–4.467)	0.2418
	No	35 (77.8)	30 (66.7)		
Positive culture	Yes	3 (10.0)	4 (9.5)	0.947 (0.196–4.582)	0.9464
	No	27 (90.0)	38 (90.5)		
Days of mechanical ventilation	Median (IQR)	5 (4–6)	8 (5–12)	n.a	0.0213
Days of non-invasive ventilation	Median (IQR)	3 (2–4)	3.5 (1–6)	n.a	0.7928
Length of hospital stay, days	Median (IQR)	13.5 (9.8–17)	15 (10–19.5)	n.a	0.1304

*CI* confidence interval, *ICU* intensive care unit, *IQR* interquartile range, *MV* mechanical ventilation, *NIV* non-invasive ventilation, *OR* odds ratio, *PCT* procalcitonin

## Discussion

The identification of patients with moderate to severe COVID-19 diseases who are at risk of deterioration and death is very important to guide the administration of appropriate treatment promptly to improve prognosis. Our study provided a comprehensive analysis of the prognostic value of serum PCT in predicting various clinical outcomes, especially for mortality and the requirement of mechanical ventilation among patients with category 4 and 5 COVID-19 diseases.

Our study results confirmed that the optimal PCT cut-point of 0.2 ng/mL is a useful biomarker to predict the risk of deterioration and death. Procalcitonin is a precursor of calcitonin which is synthesized and released by thyroid parafollicular C cells and usually remain undetectable in physiological condition, however, in the presence of inflammatory cytokines and endotoxins, it can be secreted by extrathyroidal tissues in high amounts [20].

Traditionally, an elevated PCT level is more suggestive of a bacterial infection and has long been used to guide decisions of antibiotic initiation [21]. Some studies suggested that an elevated PCT level in association with clinical deterioration of patients with COVID-19 was attributed to the secondary bacterial co-infection, which further exacerbated the primary COVID-19 infection [1, 11, 21–23]. However, the ability of PCT to accurately differentiate between bacterial and viral infection remains controversial [24]. Our study results did not identify a clear significant association between elevated PCT levels and bacterial co-infection as evidenced by the lack of positive culture yield in the majority of our patients.

The primary mechanism behind the clinical deterioration observed amongst COVID-19 patients is a supraphysiological response known as cytokine release storm. In a study by Guo et al., hypercytokinemia, especially pro-inflammatory cytokines like IL-1β, IL-6, IP-10, G-CSF, IL-8, IL-17, TNF-alpha, and IFN-gamma were seen in patients with COVID-19 and were positively associated with disease severity, multi-organ failure, and death [15, 25, 26]. The IL-6, in particular, is a useful non-specific inflammatory marker to predict disease severity [27, 28]. An increase in IL-6 and other cytokines can also trigger the increase in PCT, especially in the presence of a hyperinflammatory state, which possibly explains the positive association between PCT levels and disease severity and clinical deterioration [11, 22, 27].

Being consistent with previous studies, our study shows that a PCT level above 0.2 ng/mL is significantly associated with a higher risk of mortality, especially among severe and critically ill COVID-19 patients [1, 5, 23]. Furthermore, a meta-analysis by Huang et al. showed that an elevated PCT level was associated with increased mortality, which is in agreement with our results [29]. While our study did not find a relationship between PCT level of 0.2 ng/mL and risk of mechanical ventilation, we observed a significantly higher risk of non-invasive ventilation amongst severe and critically ill COVID-19 patients with elevated PCT levels above 0.2 ng/mL. One plausible explanation for this observation is the strategy of initiation of non-invasive ventilation, in particular high flow nasal cannula practiced in our center to reduce the rate of invasive mechanical ventilation. Several studies have found a significant reduction in invasive mechanical ventilation rates with high flow nasal cannula, which supports our strategy [30–32].

Another notable finding of our study is a longer duration of mechanical ventilation seen amongst severe and critically ill COVID-19 patients with an elevated PCT level of 0.2 ng/mL or more. This finding is very relevant, especially at the peak of this pandemic which sees a scarcity of resources like mechanical ventilators at COVID-19 treatment centers which have contributed to an increase in mortality rate [33, 34]. Furthermore, a longer duration of mechanical ventilation brings about a host of complications like ventilator-induced lung injury, major functional disabilities due to ICU acquired weakness, and cognitive impairment, which can further contribute to increased morbidity and mortality [35]. Thus, prognostication using the PCT level is important to guide rationing of medical resources and implementing relevant management strategies to minimize complications related to mechanical ventilation to minimize mortality.

## Conclusion

The results of our study clearly illustrate that an elevated serum PCT of 0.2 ng/mL or more is associated with a higher risk of mortality, NIV, and a longer duration of mechanical ventilation. Our study however did not find an association between serum PCT and risk of mechanical ventilation. On this basis, serum PCT could be effectively used as a prognostic biomarker to predict mortality, the requirement of NIV, and duration of mechanical ventilation in severe and critical COVID-19 patients. However, to better understand the implications of these results, further large adequately powered studies could be done to look into the efficacy of various COVID-19 treatment strategies by looking at its ability to reduce serum PCT, which could signify a reduction in risk of mortality, requirement of NIV and a shorter duration of mechanical ventilation.

## Data Availability

The datasets generated and analyzed during the current study are available in the GitHub repository, https://github.com/william81/procalcitonin

## Abbreviations

COVID-19: Coronavirus disease 2019
PCT: Procalcitonin
OR: Odds ratio
CI: Confidence interval
p: P-value
WHO: World Health Organisation
ICU: Intensive care unit
CRP: C-reactive protein
LDH: Lactate dehydrogenase
PCR: Polymerase chain reaction
URT: Upper respiratory tract
IM: Immunoassay
MREC: Medical Research Ethics Committee
ROC: Receiver operative characteristic
IBM: International Business Machines Corporation
SPSS: Statistical Package for the Social Sciences
SD: Standard deviation
HIV: Human immunodeficiency virus
AIDS: Acquired immunodeficiency syndrome
NIV: Non-invasive ventilation
IQR: Interquartile range
CT scan: Computed tomography
AUC: Area under curve
TP: True positive
FN: False negative
FP: False positive
TN: True negative
SPO_2_: Oxygen saturation
GCS: Glasgow Coma Scale
WBC: White blood cells
ANC: Absolute neutrophil count
DM: Diabetes mellitus
RR: Respiratory rate
MV: Mechanical ventilation
HPT: Hypertension
RA: Room air
IL: Interleukin
IP-10: Interferon gamma-induced protein 10
G-CSF: Granulocyte colony-stimulating factor
TNF-alpha: Tumour Necrosis Factor alpha
IFN-gamma: Interferon gamma
